# Protection from lethal challenge in a neonatal mouse model by circulating recombinant form coxsackievirus A16 vaccine candidates

**DOI:** 10.1099/vir.0.063560-0

**Published:** 2014-05

**Authors:** Jingliang Li, Junliang Chang, Xin Liu, Jiaxin Yang, Haoran Guo, Wei Wei, Wenyan Zhang, Xiao-Fang Yu

**Affiliations:** 1 First Hospital of Jilin University, Institute of Virology and AIDS Research, Changchun, Jilin Province, PR China; 2 Department of Molecular Microbiology and Immunology, Johns Hopkins Bloomberg School of Public Health, 615 N. Wolfe Street, Baltimore, MD 21205, USA

## Abstract

Circulating coxsackievirus A16 (CA16) is a major cause of hand, foot and mouth disease (HFMD) in South-east Asia. At present, there is no vaccine against CA16. Pathogenic animal models that are sensitive to diverse circulating CA16 viruses would be desirable for vaccine development and evaluation. In this study, we isolated and characterized several circulating CA16 viruses from recent HFMD patients. These CA16 viruses currently circulating in humans were highly pathogenic in a newly developed neonatal mouse model; we also observed and analysed the pathogenesis of representative circulating recombinant form CA16 viruses. An inactivated CA16 vaccine candidate, formulated with alum adjuvant and containing submicrogram quantities of viral proteins, protected neonatal mice born to immunized female mice from lethal-dose challenge with a series of CA16 viruses. Further analysis of humoral immunity showed that antibody elicited from both the immunized dams and their pups could neutralize various lethal viruses by a cytopathic effect *in vitro*. Moreover, viral titres and loads in the tissues of challenged pups in the vaccine group were far lower than those in the control group, and some were undetectable. This lethal-challenge model using pathogenic CA16 viruses and the vaccine candidates that mediated protection in this model could be useful tools for the future development and evaluation of CA16 vaccines.

## Introduction

Coxsackievirus 16 (CA16) and enterovirus 71 (EV71) both belong to the *Picornaviridae*, a family of single-stranded positive-sense RNA viruses, and can cause hand, foot and mouth disease (HFMD) with various neurological symptoms (Hagiwara *et al.*, 1978). HFMD has become a serious public health problem in South-east Asia, with periodic large epidemics occurring in recent decades. Past virological investigations and studies have focused mostly on EV71 because of its association with severe complications involving the central nervous system and significant mortality (Chen *et al.*, 2007; Qiu, 2008). Outbreaks of HFMD due to CA16 occurred in England in 1994 (Bendig & Fleming, 1996), Taiwan from 1999 to 2006 (Wu *et al.*, 2010), Singapore from 2001 to 2007 (Ang *et al.*, 2009) and South India from 2005 to 2008 (Vijayaraghavan *et al.*, 2012). Moreover, accumulating evidence has demonstrated that CA16 infection also causes severe neurological complications (Yen *et al.*, 2009) and death (Wang *et al.*, 2004; Wright *et al.*, 1963). Also of concern is the fact that EV71 and CA16 can co-infect humans and exist simultaneously in the same host (Yan *et al.*, 2012), potentially allowing the formation of new recombinants of CA16 and EV71 (Zhao *et al.*, 2011; Li *et al.*, 2011). As an EV71 vaccine alone is not sufficient to prevent HFMD outbreaks, both EV71 and CA16 should be targeted for vaccine development so as to provide broad and effective protection against HFMD.

At present, the inactivated EV71 vaccines developed in mainland China have completed phase I and II clinical trials (Meng *et al.*, 2012). However, it is not clear whether CA16 is sufficiently immunogenic to elicit broad protective immune responses and no CA16 vaccine has yet entered clinical trials. The newborn mouse model of lethal EV71 infection (Chen *et al.*, 2011; Wu *et al.*, 2001, 2007) has played a critical role in the development and evaluation of EV71 vaccines. Clearly, the development of an analogous CA16 animal model for the evaluation of vaccine candidates is needed urgently.

Recently, lethal CA16 challenge strains in neonatal mouse systems have been reported (Liu *et al.*, 2012; Mao *et al.*, 2012). However, the availability of viral isolates of currently circulating CA16 viruses from HFMD patients is very limited and it is still unknown if these viruses are pathogenic in the mouse model. In this study, we isolated and identified multiple circulating CA16 viruses from recently infected HFMD patients. These CA16 viruses produced a lethal pathogenic infection in our newborn mouse model. More importantly, we observed protection from lethal challenge in these neonatal mice by circulating recombinant CA16 vaccine candidates. The lethal-challenge model using pathogenic CA16 viruses and the vaccine candidates that mediated protection in this model could serve as useful tools for the future development and evaluation of CA16 vaccines.

## Results

### Circulating recombinant CA16CC024 viruses produced dose-related disease symptoms and mortality in neonatal mice

To establish animal models for the study of current circulating CA16 viruses, we isolated several CA16 viruses from recently infected HFMD patients. These viruses were identified genetically as recombinant forms of CA16 virus. CA16CC024 was first evaluated for pathogenesis in newborn mice because viral infection with this strain has been associated with viral meningitis in HFMD patients. Various doses of CA16CC024 were injected intracerebrally into 1-day-old mice as reported previously (Mao *et al.*, 2012). Clinical scores and the mortality rate of newborn mice were examined.

**Fig. 1.**
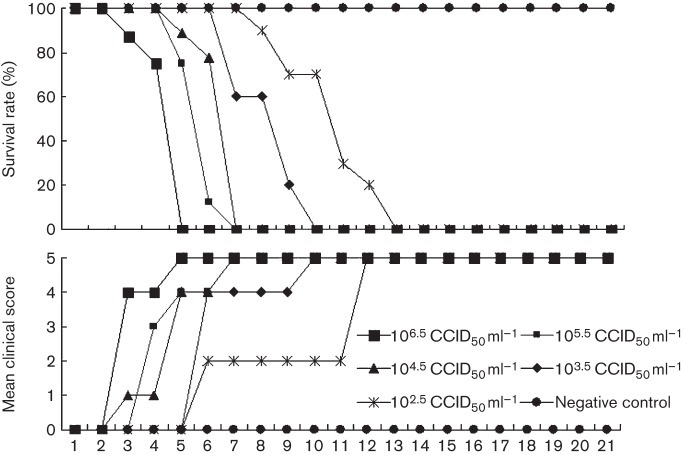
Circulating CA16CC024 virus resulted in dose-related disease and mortality in newborn mice. One-day-old ICR mice were intracerebrally inoculated with increasing doses of CA16CC024, from 10^2.5^ to 10^6.5^ CCID_50_ ml^−1^ (10 µl). Survival rates and clinical scores were then monitored and recorded daily after infection. Control mice were given medium instead of virus suspension. Each group contained 8–10 mice. The results are from three independent experiments. Varying grades of clinical disease are identified as: 0, healthy; 1, lethargy and inactivity; 2, wasting; 3, limb-shake weakness; 4, hind-limb paralysis; 5, moribund or dead.

The mice that were infected with the highest dosage of CA16CC024 [10^6.5^ 50% cell culture infective dose (CCID_50_) ml^−1^] became sick on day 3 post-infection, had a mean clinical score of grade 4 and mortality reached 100 % by day 6 (Fig. 1). As expected, the mice that were injected with medium alone (negative control group) showed a clinical score of grade 0 and had a 100 % survival rate. The mice challenged with CA16CC024 at 10^5.5^, 10^4.5^ or 10^3.5^ CCID_50_ ml^−1^ showed increasing morbidity on days 3–6 (clinical score, grades 1–5), with a 100 % mortality rate on days 7, 7 and 10, respectively (Fig. 1). The mice that were infected with the lowest dose of CA16CC024, 10^2.5^ CCID_50_ ml^−1^, showed increasing morbidity on day 6 (grade 2) and mortality reached 100 % by day 13. Thus, the symptoms and mortality rate increased steadily as the dose increased.

### Pathological analysis of CA16 strains in neonatal mice

**Fig. 2.**
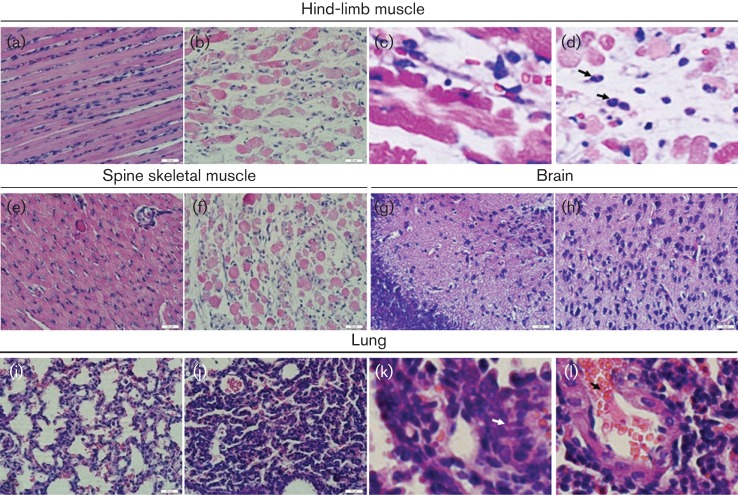
Pathological analysis of CA16-infected neonatal mice. One-day-old ICR mice were intracerebrally inoculated with medium or CA16CC024. No histological change was observed in the hind-limb muscle (a) or spine skeletal muscle (e) of the non-infected control mice. Infected mice (grades 4–5) exhibited severe necrosis, including muscle bundle fracture, dissolution of muscle fibre cells, nuclear shrinkage and swelling (b), and inflammatory cell infiltration (arrow) (c, d). Infected mice (grades 4–5) showed loose muscle fibres in the juxtaspinal skeletal muscle (f). No histological change was observed in the brains of the non-infected control mice (g) or CA16-infected mice (h). No histological changes were observed in the lungs of the non-infected control mice (i). Mice infected with CA16CC024 exhibited severe alveolar shrinkage (j), a few areas of scattered pulmonary fibrosis (k), a large number of red cells (j, arrow), and vascular dilatation and congestion (j) in the lung tissue. Magnification: a, b, e–j: ×400 (scale bars, 20 μm); c, d, k, l: ×2000. The results are representative of three independent experiments.

To understand the pathogenic effects of CA16CC024 that may be related to the death of the newborn mice, we performed a systematic pathological analysis of various tissues, including brain, lung, spine skeletal muscle, hind-limb muscle, liver, kidney, spleen, heart and intestine, from infected mice with a grade 5 clinical score that were challenged with CA16CC024 at 10^3.5^ CCID_50_ ml^−1^. We observed that the hind-limb muscle fibres exhibited severe necrosis, including muscle bundle fracture, dissolution of muscle fibre cells, and swelling and shrinkage of the nuclei (Fig. 2b), when compared with those of non-infected mice (Fig. 2a). Infiltration of inflammatory cells into the muscle tissues of the sick mice was also detected (Fig. 2c, d). Moreover, severe necrosis was observed in the spine skeletal muscle fibres of mice infected with CA16CC024 (Fig. 2f) when compared with the negative controls (Fig. 2e). No obvious pathological changes were found in the brain (Fig. 2h), liver, kidney, spleen, cardiac muscle or intestine (data not shown). Interestingly, CA16CC024 infection of the neonatal mice caused obvious lung tissue lesions, including severe alveolar shrinkage (Fig. 2j), a few scattered areas of pulmonary fibrosis (Fig. 2k), pulmonary oedema, and vascular dilatation and congestion (Fig. 2l). In the negative control group, no pathological changes were found in the examined tissues (Figs 2a, e, g, i). These results demonstrated that CA16CC024 had a strong tropism for the muscle and lung tissue, and led to severe lesions in these tissues.

### Detection of viral antigen in selected tissues of CA16CC024-infected mice

**Fig. 3.**
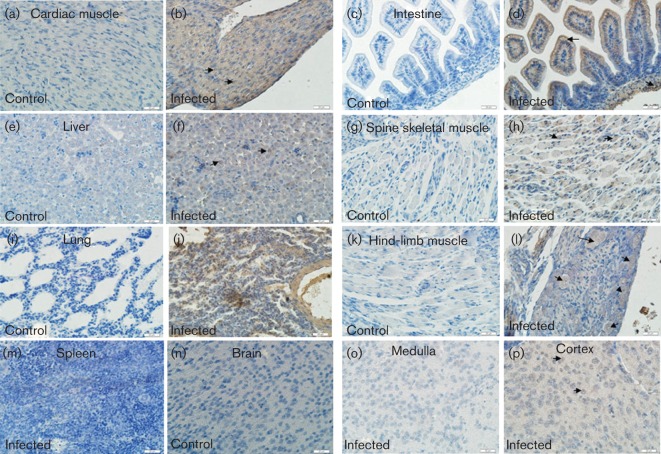
Viral antigen expression in tissues from CA16CC024-infected neonatal mice. The tissues were obtained on day 5 after intracerebral inoculation with medium or CA16CC024 at 10^3.5^ CCID_50_ ml^−1^. Samples were embedded and frozen in liquid nitrogen, then fixed in 3.7 % paraformaldehyde and treated with hydrogen peroxide (2.5 %). Rabbit anti-CA16 antibody and HRP-conjugated anti-rabbit IgG antibody were used for immunohistochemical staining. Magnification:×400 (scale bars, 20 μm). The viral antigen was exhibited in the heart (b), intestine (d), liver (f), spinal skeletal muscle (h), lung (j), hind-limb muscle (l) and cortex of the brain (p) of infected mice, but not in the spleen (m) or brain medulla (o). In contrast, no viral antigen was detected in the heart (a), intestine (c), liver (e), spine skeletal muscle (g), lung (i), hind-limb muscle (k) or brain (n) of non-infected mice. The results are representative of three independent experiments.

To further investigate the relationship between the pathogenesis of CA16CC024 and its replication in tissues, we examined the expression of the CA16CC024 antigen in the associated tissues of infected mice. We observed high expression of the viral antigen in the muscle of the spinal region (Fig. 3h), lung (Fig. 3j) and hind limb (Fig. 3l), all of which were areas in which pathological changes occurred (Fig. 2). Viral antigens were also observed in the cardiac muscle (Fig. 3b), intestine (Fig. 3d) and liver (Fig. 3f), although pathological changes were not observed in these tissues. Weaker antigen signals were observed in the cortex of the brain (Fig. 3p). Consistent with the lack of any pathological changes in these tissues, no viral antigen was detected in the spleen (Fig. 3m) or brain medulla (Fig. 3o) of the infected mice. As expected, no viral antigen was detected in non-infected mice in any of the tissues examined (Figs 3a, c, e, g, i, k, n), indicating that the detection of viral antigens was specific. These results demonstrated that CA16CC024 could induce widespread infection, but only triggered tissue-specific pathogenesis in infected mice.

### Kinetics of viral replication in various tissues of CA16CC024-infected neonatal mice

**Fig. 4.**
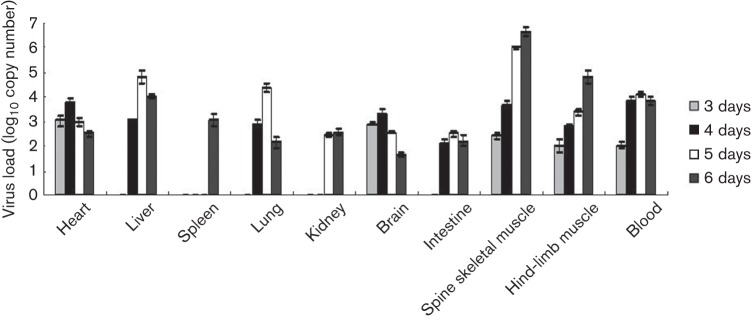
Viral load variations in various tissues of CA16CC024-infected mice at different time points. One-day-old ICR mice were inoculated intracerebrally with 10 µl CA16CC024 at 10^3.5^ CCID_50_ ml^−1^. Virus loads were assessed by real-time quantitative reverse transcriptase-PCR in samples of the intestine, lung, liver, hind-limb muscle, brain, spleen, kidney, heart, spine and blood from the infected mice. Samples were collected at the times indicated. The results represent the mean virus loads [log_10_ copies (mg tissue)^−1^ or log_10_ copies (ml blood)^−1^]±sd (three mice per group, repeated three times).

To further understand the replication and distribution of CA16 viruses in infected mice, we measured the viral loads in various tissues from infected mice at different times post-infection. Viral loads were detected in the heart, brain, spine skeletal muscle, hind-limb muscle and blood at day 3 after infection in CA16CC024-infected mice (Fig. 4). At 4 days after infection, the virus was detected in more of the tissues examined, but not in the spleen or kidney. Relatively high viral loads were observed in the heart (10^3.76^ copies mg^−1^), brain (10^3.30^ copies mg^−1^), spine skeletal muscle (10^3.69^copies mg^−1^) and blood (10^3.83^ copies ml^−1^). From day 4 or 5, the viral loads in the liver, lung, kidney, intestine, spine skeletal muscle, hind-limb muscle and blood all increased steadily (Fig. 4). On day 6 after infection, muscle tissue carried the highest viral load. No viral loads were detected in the same tissues of non-infected mice, demonstrating that the viral load detection was specific.

In general, viral replication reached a peak on day 4 or 5 after infection in affected tissues, including the heart, liver, lung, intestine and blood (Fig. 4). However, viral loads continued to increase in the spine skeletal muscle and hind-limb muscles (Fig. 4). It is worth noting that no virus was detected in the spleen until day 6. Detection of viral loads at 5 days post-infection is consistent with our immunohistochemical analysis (Fig. 3).

### Maternal immunization with an inactivated CA16 vaccine candidate provided protection against lethal virus challenge in neonatal mice

**Fig. 5.**
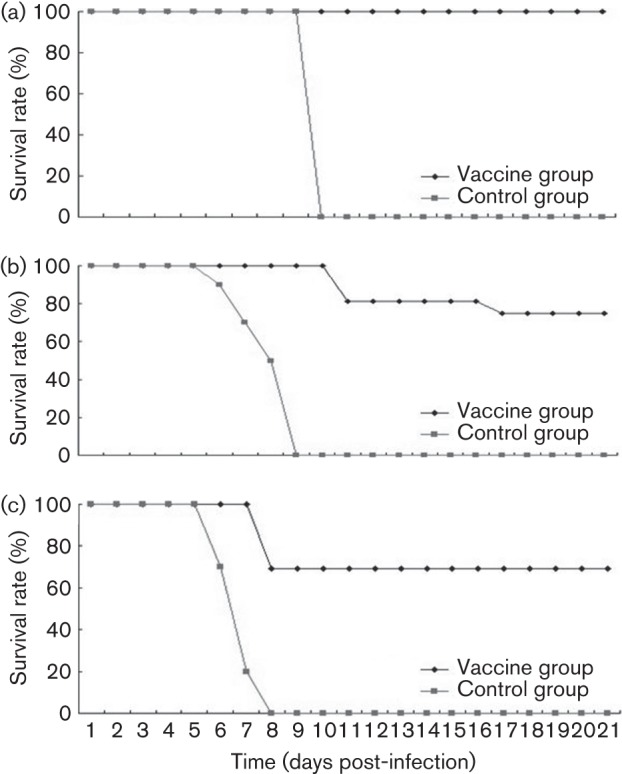
Maternal immunization with inactivated CA16CC024 protected newborn mice from CC024 lethal challenge. Adult female ICR mice were immunized twice at 2 week intervals and allowed to mate with male mice after the first injection. After delivery, pups (*n* = 8–10) were challenged with the lethal CA16CC024 strain at (a) 10^3.5^, (b) 10^4.5^ or (c) 10^5.5^ CCID_50_ ml^−1^. Survival rates and clinical scores were monitored for 21 days post-challenge.

As the CA16CC024 strain induced the death of neonatal mice, we wanted to know if the CA16CC024 vaccine candidate could protect the neonatal mice born to immunized dams from lethal challenge by autologous CA16CC024 viruses. The female mice were immunized intraperitoneally twice at 2 week intervals with inactivated CA16CC024 virus formulated with alum (PBS was used as a negative control). They were allowed to mate with the male mice after the first injection. After delivery, 1-day-old mice were challenged with 10 µl CA16CC024 at 10^5.5^, 10^4.5^ or 10^3.5^ CCID_50_ ml^−1^ (Fig. 8a). The neonatal mice born to unimmunized mice (PBS) started to die on day 6 after virus inoculation and mortality reached 100 % by day 10 after challenge (Fig. 5). One-day-old mice born to dams immunized with inactivated CA16CC024 had survival rates of 100, 75 and 69.23 % when challenged with CA16CC024 at 10^3.5^, 10^4.5^ and 10^5.5^ CCID_50_ ml^−1^, respectively (Fig. 5). In addition, these mice showed no severe clinical symptoms (data not shown).

### Divergent CA16 viruses induced lethal infection of neonatal mice, which could be prevented by maternal immunization with inactivated CA16 vaccine candidate

**Fig. 6.**
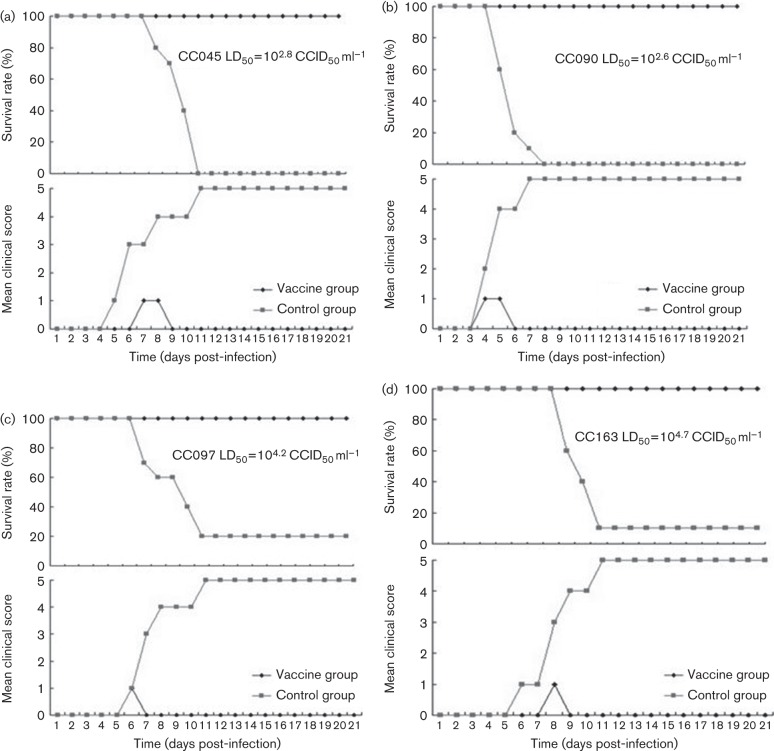
Maternal immunization with inactivated CA16CC024 virus protected newborn mice from lethal challenge with (a) CC045, (b) CC090, (c) CC097 and (d) CC163 (d). Adult female ICR mice were immunized twice at 2 week intervals and allowed to mate with male mice after the first injection. After delivery, pups (*n* = 8–10) were challenged with the lethal strain. Clinical scores and survival rates were monitored for 21 days post-challenge.

**Fig. 7.**
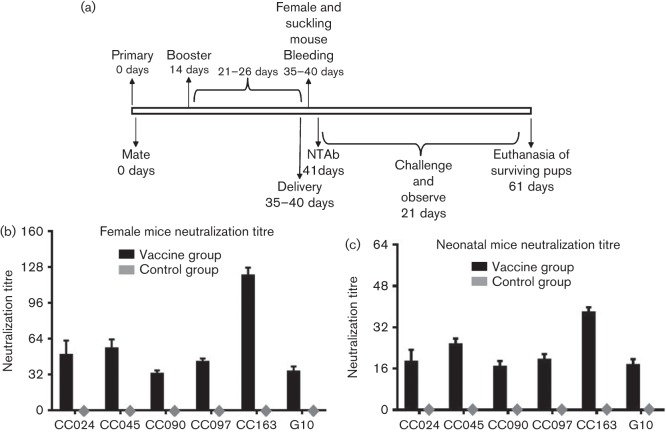
Neutralization titres of anti-CA16 sera in immunized dams and pups. (a) Diagram of the experimental procedure. (b, c) Adult female ICR mice were immunized and examined for NTAb titres as described in Methods. The sera were collected from three dams and their pups (8–10 mice per group, mixed) after delivery. The mean neutralization titres against various CA16 strains were measured as described in Methods. The values for the negative control groups were all <2.

To determine whether our neonatal-lethal model in mice was sensitive to other circulating recombinant CA16-related viruses, we examined the pathogenic effect of four unrelated CA16 viruses isolated from HFMD patients. The female mice were immunized and allowed to mate with the male mice as described in Methods (Fig. 7a). When the 1-day-old mice were challenged with CC045 at ~100 LD_50_ (Fig. 6a), CC090 at 10 LD_50_ (Fig. 6b), CC097 at 2 LD_50_ (Fig. 6c) or CC163 at 8 LD_50_ (Fig. 6d), all the mice developed disease symptoms, with a mortality rate of 80–100 %. In contrast, all the 1-day-old mice born to dams immunized with inactivated CA16CC024 showed a 100 % survival rate, although a few mice exhibited disease symptoms with a clinical score of grade 1, but they recovered quickly (Fig. 6). These data suggested that immunization of female adult mice using the inactivated CA16CC024 vaccine candidate could significantly protect the neonatal mice against heterologous lethal viral challenge.

### CA16CC024 vaccine candidate elicited a protective humoral response in immunized female mice and their pups

**Fig. 8.**
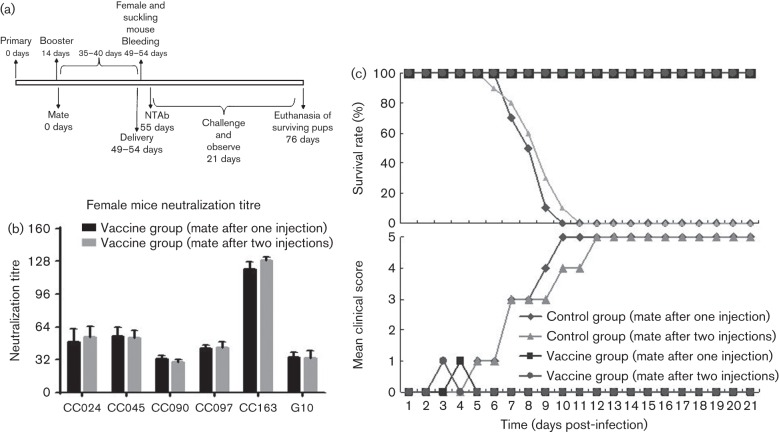
Protective efficacy of mating female mice after the first or second injection. (a) Diagram of the experimental procedure. (b) Adult female mice were immunized intraperitoneally twice at 2 week intervals with inactivated CA16CC024 viruses or PBS formulated with alum. One group containing three female mice immunized with inactivated CC024 vaccine was allowed to mate with the male mice after the second injection and sera were collected for measurement of NTAb titres as shown in diagram (a) after delivery. The mean neutralization titres against various CA16 strains were measured and compared with those generated using a different procedure, as described in Fig. 7. (c) After delivery, pups (*n* = 8–10) born to immunized female mice mated with male mice after the first or second injection were challenged with the lethal CA16CC024 strain at 10^3.5^ CCID_50_ ml^−1^ (equal to 100 LD_50_). Survival rates and clinical scores were monitored for 21 days post-challenge.

The inactivated CA16CC024 vaccine candidate was able to provide protection against CA16CC024 and other heterologous CA16 viruses in neonatal mice born to immunized female mice. We next examined whether inactivated CA16CC024 was able to induce protective neutralizing antibody (NTAb) responses in immunized female mice and their pups. The female mice were immunized and allowed to mate with the male mice as indicated (Fig. 7a). After delivery, serum samples were collected from three immunized dams and their pups at day 1 (Fig. 7a). NTAb titres against various CA16 strains (CC024, CC045, CC090, CC097, CC163 and G10) were measured as reported previously (Mao *et al.*, 2012). The NTAb titres of immunized female mice reached 1/32 to 1/128 (Fig. 8b). The NTAb titres of non-immunized control mice were below the limit of detection (Fig. 7b). The pups born to the immunized female mice, but not those born to non-immunized mice, also had high titres of NTAb (Fig. 7c). NTAb titres against the most divergent virus, CA16 (G10), were also detected.

Previous reports related to the protective efficacy of the EV71 or CA16 vaccine in the mouse model have involved mating the female mice after one immunization (Chen *et al.*, 2011; Mao *et al.*, 2012). We compared the strategies of mating the female mice after one or two immunizations. The female mice were immunized intraperitoneally twice at 2 week intervals with inactivated CA16CC024 vaccine. They were allowed to mate with the male mice after the second injection (Fig. 8a). After delivery, serum samples were collected from immunized female mice on day 1. NTAb titres against CA16 strains CC024, CC045, CC090, CC097, CC163 and G10 were measured. Similar NTAb titres against the various CA16 strains were detected in female mice after the first or second injection (Fig. 8b). Moreover, immunization procedures involving mating the female mice after the first or second injection also protected the neonatal mice from lethal-dose challenge with CC024 viruses (Fig. 8c).

### CA16 viral replication was suppressed in pups born to the vaccine-immunized mice

To better understand the mechanism protecting neonatal mice in the vaccine group from lethal-dose challenge, we compared the viral titres in the tissues of pups born to immunized or control female mice at different time points after virus challenge. Subsequent experiments showed that the virus titres (Fig. S1, available in the online Supplementary Material) in the heart, liver, lung, brain, intestine, spine skeletal muscle, hind-limb muscle and blood of the neonatal mice born to immunized dams were mostly undetectable, as compared with those of the control group. Thus, the neutralization antibodies in pups born to immunized dams were associated with viral suppression and escape from lethal challenge.

## Discussion

In the current study, we evaluated multiple CA16 viruses in a lethal-challenge model using neonatal mice. These CA16 virus strains were isolated in 2010 from recently hospitalized HFMD patients in the north-east region of China who had severe or mild clinical symptoms. These patients were epidemiologically unrelated and from Jilin Province, which has a population of 27.5 million people and covers an area of 187 400km^2^. The new clinical isolates used in this study are closely related to other CA16 strains isolated in China and reported by other groups (Fig. S2). The CA16 strains isolated in China have been characterized as circulating recombinant CA16-related viruses that are different from the prototype CA16-G10 (Zhao *et al.*, 2011). Even in the most immunogenic structural protein region (VP1), there are differences of >8 % in the amino acid sequences of CC024 (vaccine strain) and CA16 G10 (prototype CA16).We observed that the neonatal mouse model was sensitive to lethal viral infection with diverse circulating CA16 strains. Therefore, this mouse model could facilitate future study of viral pathogenesis as well as vaccine development.

We discovered that CA16 viruses generated many symptoms in the neonatal mice, such as lethargy, hind-limb paralysis and severe lesions in the lung tissue, including pulmonary oedema and haemorrhage. Interestingly, these findings were consistent with observations in patients infected with CA16 viruses (Legay *et al.*, 2007). It has also been reported that humans infected fatally with EV71 viruses exhibit pulmonary oedema, damage to lung tissue and inflammatory cell aggregation in the lung tissue (Kao *et al.*, 2004). Lung tissue damage appears to be unique to our study and has not been observed in other CA16 pathogenic mouse models (Chan & AbuBakar, 2005; Mao *et al.*, 2012). Future study will be required to determine whether these pathogenic effects on lung tissues are a unique feature of our CA16 strains.

Immunohistochemical staining revealed the presence of viral antigen in the muscle and lung tissues. These results were consistent with the results of viral load measurement in the tissues. Furthermore, high levels of viral replication were correlated with pathological observations in the muscles and lung tissue (Fig. 2). The expression of viral antigen could also be detected in many other tissues of the infected mice by immunohistochemical staining and these results were further confirmed by viral load measurement in the tissues. However, we observed the most obvious lesions in the spine and hind-limb muscles as well as the lung tissue. Thus, the replication of CA16 viruses in some tissues did not induce apparent pathological changes, suggesting that the pathogenicity of CA16 virus infection is tissue-specific.

Data presented in Figs 2 and 3 contain several novel observations not seen in the report of Mao *et al.* (2012). (1) In the pathology analysis (Fig. 2), we observed unique changes in the lungs of infected mice. (2) Unlike Mao *et al.* (2012), we did not find detectable lesions in the brain or cardiac muscle. (3) Most importantly, in the immunohistochemical analysis (Fig. 3), we detected virus antigen expression in cardiac muscle – a finding that had not been reported previously and was consistent with the clinical features of certain CA16-infected patients. (4) The viral strain used in our study (north-east China, 2010) is different from that used in the Mao *et al.* (2012) study (Beijing, China, 2008). It would be interesting to investigate further the differences in the viral genome sequences or viral proteins that led to the distinct observations in the two studies.

Human scavenger receptor B2 (SCARB2) has been identified as a receptor for EV71 and CA16 (Yamayoshi *et al.*, 2009). EV71 cannot infect efficiently non-sensitive mouse L929 cells unless human SCARB2 is also introduced. P-selectin glycoprotein ligand-1 (PSGL-1) has also been shown to serve as a cellular receptor for EV71 (Nishimura *et al.*, 2009) and is expressed mainly in lymphoid cells. In the present study, we observed that multiple CA16 virus strains could infect the neonatal mice and replicate in various mouse tissues. As mouse PSGL-1 and SCARB2 are not expected to mediate efficient replication of CA16 viruses in these mice, our study raises the question of whether additional cellular receptors contribute to CA16 infection in our mouse model.

CA16 and EV71 infection are both responsible for widespread HFMD and present serious public health problems in the Asia–Pacific region (Ho *et al.*, 1999; Ma *et al.*, 2011; Yang *et al.*, 2011). As no effective anti-CA16 or anti-EV71 drug is available, developing effective vaccines against CA16 and EV71 is the best strategy for controlling the disease. For vaccine evaluation, an *in vitro* cellular cytopathogenic effect (CPE) method is usually used to detect the cross-neutralization activity of vaccine candidates against various intratypic or intertypic subtype viruses. Animal model systems can provide alternative and perhaps even better ways to evaluate the immunogenicity and protective efficacy of candidate vaccines. The ability of diverse CA16 viruses to produce the death of newborn mice in this study provides an opportunity to test vaccine candidates for cross-protection. Indeed, we have demonstrated that a CA16 vaccine candidate offered broad protection against lethal challenge with various viruses in this mouse model. Moreover, we found that female mice immunized with the inactivated CA16 vaccine candidate, as well as their pups, showed high NTAb titres against homologous and heterologous CA16 viruses. The detection of NTAb titres in the newborn mice was correlated with suppressed viral replication in these mice. Furthermore, NTAb titres were also detected against the most divergent CA16 (G10) virus. If similar protective immune responses can be induced in humans, a vaccine against CA16 may offer hope for controlling CA16-induced HFMD in children.

## Methods

### 

#### Cells and viruses.

Vero cells (CCL-81; American type Culture Collection) were grown in modified Eagle’s medium (MEM) supplemented with 10 % FBS and 3 % l-glutamine at 37 °C with 5 % CO_2_. The CA16 virus was isolated from throat swabs of Changchun HFMD patients in 2010. The viral samples were diluted in MEM medium and filter-sterilized using a 0.22 µm filter (Millipore) before being used to infect Vero cells. Viruses were harvested and continuously passage after the observation of a CPE. CC024 was used at passage 9, and CC045, CC090, CC097 and CC163 were used at passage 6 in this study. The viral titre was determined in Vero cells according to the Reed–Muench formula.

#### Neutralization assay.

The neutralization titres were determined by the TCID_50_ reduction assay in Vero cells. Serum samples were serially diluted by twofold steps in MEM and various CA16 strain stocks were diluted to a working concentration of 100 TCID_50_ ml^−1^. Subsequently, 50 µl of each diluted serum sample was mixed with 50 µl of various CA16 solutions. The mixtures were added to 96-well plates and incubated at 37 °C for 2 h. Following the incubation, 100 µl Vero cells (2×10^5^ ml^−1^) was seeded onto the 96-well plates for infection and cultured at 35 °C with 5 % CO_2_. At 7 days post-infection, the infected cells were observed under a microscope for the presence of a CPE. Neutralization titres were determined as the highest serum dilution that could prevent the appearance of a CPE in >50 % of the cell cultures (performed in quadruplicate).

#### Neonatal mouse challenge test.

Care and use of the animals in the experimental procedures were approved by the Office of Laboratory Animal Management of Jilin University. One-day-old specific pathogen-free (SPF) ICR neonatal mice (weight 1.8–2.0 g, provided by the Experimental Animal Center, College of Basic Medicine, Jilin University) were divided randomly into different experimental groups, with three litters per group and 8–10 neonatal mice per litter. The neonatal mice were inoculated intracerebrally with 10-fold serial dilutions of different virus strains or MEM medium, respectively. The grade of clinical disease was scored as follows: 0, healthy; 1, lethargy and inactivity; 2, wasting; 3, limb-shake weakness; 4, hind-limb paralysis; 5, moribund or dead. Body weight, activity, and the occurrence of limb paralysis, morbidity and death were recorded for 21 days post-infection. The control mice were healthy throughout the experiments. The LD_50_ was calculated by the Reed–Muench formula.

#### Histopathological and immunohistochemical analysis.

Six mice were sampled: three dying mice from the experimental group (with obvious pathological features) and three normal mice from the MEM control group. Various tissue samples from the organs of the infected or non-infected mice, including brain, lung, spinal muscle, hind-limb muscle, liver, kidney, spleen, heart and intestine, were fixed in 10 % formalin for 3–5 days. All tissues were dehydrated through an ethanol gradient and then embedded in paraffin before obtaining 4 µm sections for further haematoxylin and eosin staining. Histopathological analysis of the tissues was performed under a light microscope.

For immunohistochemical analysis, tissue samples were embedded in optimal cutting temperature compound and frozen in liquid nitrogen. The frozen tissue samples were then cut into 4 µm sections, placed on poly-l-lysine-coated glass slides and fixed in 3.7 % paraformaldehyde. The endogenous peroxidase activity of the tissues was inhibited by treatment with hydrogen peroxide (2.5 %). CA16 antigen was captured by rabbit anti-CA16 polyclonal antibody (made in our laboratory) and detected by a Streptavidin-Peroxidase Anti-Rabbit IgG kit (Maixin), followed by colour development with diaminobenzidine for detection of the antigen–antibody reaction.

#### Viral loads and viral titres in neonatal mice tissues post-challenge.

After intracerebral inoculation with CA16 virus or uninfected culture medium, three experimental neonatal mice and three control neonatal mice were subjected to viral load and viral titre assays. Blood and tissue samples from the experimental group were collected on days 1, 2, 3, 4, 5 and 6 post-infection; for uninfected mice, they were collected on day 1 before challenge. All tissues, including heart, liver, spleen, lung, kidney, brain, intestine, spine skeletal muscle and hind-limb muscle, were weighed individually and homogenized in sterile PBS, disrupted by freeze–thawing, and centrifuged.

In order to detect the viral loads and viral titres of challenged mice in the immunized group, half of the samples were treated with TRIzol (Invitrogen) for RNA extraction and viral load determination by real-time PCR as described previously (Sarkis *et al.*, 2006). The other half of the samples were used for viral titre determination. Viral loads were determined by real-time PCR and expressed as log_10_ copies (mg tissue)^−1^ or log_10_ copies (ml blood)^−1^. The viral titre was determined in Vero cells according to the Reed–Muench formula.

#### RNA extraction and real-time PCR.

For quantitative real-time PCR, viral RNA was extracted from fresh tissue homogenates from mice using TRIzol, and cDNA was generated using a High-Capacity cDNA Reverse Transcription kit (Applied Biosystems) and oligo-d(T)_18_ primers according to the supplier’s instructions. Primers, designed using the VP1 conserved region sequences of CA16, were as follows: CA16-F1: CATGCAGCGCTTGTGCTT; CA16-F2: CATGCAACGACTGTGCTTTC; CA16-R1: CACACAATTCCCCCGTCTTAC; CA16-R2: CATAATTCGCCCGTTTTGCT. The SYBR Green-based real-time reverse transcription-PCR was carried out on an Mx3005P (Agilent Technologies Stratagene) using the dsDNA-binding dye method with SYBR Green PCR Master Mix (Applied Biosystems). Each 20 µl reaction mixture contained 10 µl SYBR Premix; 0.2 µl (10 µM) each of F1, R1, F2 and R2; 7.2 µl double-distilled H_2_O; and 2 µl cDNA templates. Cycling conditions were as follows: 50 °C for 2 min, then 95 °C for 10 min, followed by 50 cycles consisting of 95 °C for 15 s and 60 °C for 1 min. The melting curve analysis was done at 90 °C for 1 min, then at 55 °C for 30 s and 95 °C for 30 s.

The copy number of the target cDNA in the quantitative real-time PCR was determined by using a standard curve of 10-fold serial dilutions of non-linearized plasmid DNA containing the target VP1 sequence (ranging from 10^2^ to 10^9^ copies). Absolute RNA copy numbers were calculated by using standard dilution curves of plasmids containing the target sequence. The sensitivity of the assay or limit of detection was determined to be the lowest copy number that was amplified consistently within the linear portion of the standard curve.

#### CC024 vaccine preparation, serum antibody neutralization and protective efficacy against lethal challenge with various CA16 strains.

The infected Vero cells were harvested, centrifuged at 4500 ***g*** for 30 min and concentrated 20-fold by ultrafiltration. The viruses were inactivated with 1/4000 formalin at 37 °C for 120 h. After inactivation, purification was performed on a Sepharose 4 Fast Flow column and the amount of viral proteins was determined. Prior to immunization, partially purified inactivated CA16CC024 vaccine candidate or similarly prepared negative control samples in PBS were mixed with alum and 0.5 ml (10 µg ml^−1^) of the antigen/alum mixture was used for each immunization.

Female ICR mice, 8 weeks of age (*n* = 3, each group), were injected intraperitoneally with inactivated CA16CC024 vaccine or negative control PBS at 2 week intervals. The dosage of inactivated vaccine corresponded to 10 µg CA16CC024. The mice were allowed to mate 1 h after the first injection. We also performed another immunization procedure, which involved mating with male mice after the second injection. About 35–40 days after mating, pups were born and intracerebrally challenged with 10-fold diluted CC024 and various lethal CA16 strains at different challenge doses on day 1. Clinical symptoms and death of the challenged newborn mice were monitored for 21 days. At the same time, three female mice and their pups in the vaccine and PBS groups were immediately euthanized, and sera were collected for NTAb after delivery. Neutralization titres represent the geometric mean antibody count, and were determined as the highest serum dilution that could prevent the appearance of a CPE in 50 % of the cell cultures.

#### Statistical analysis.

The values for the antibody titres, viral loads and clinical scores were analysed using a non-parametric one-way ANOVA test. Survival rates were evaluated by a log-rank test. Results are expressed as mean±sem. *P*<0.05 was considered significant.
